# Hepatocyte cultures: From collagen gel sandwiches to microfluidic devices with integrated biosensors

**DOI:** 10.1063/5.0058798

**Published:** 2021-10-14

**Authors:** Jose M. de Hoyos-Vega, Hye Jin Hong, Gulnaz Stybayeva, Alexander Revzin

**Affiliations:** Department of Physiology and Biomedical Engineering, Mayo Clinic, Rochester, Minnesota 55902, USA

## Abstract

Hepatocytes are parenchymal cells of the liver responsible for drug detoxification, urea and bile production, serum protein synthesis, and glucose homeostasis. Hepatocytes are widely used for drug toxicity studies in bioartificial liver devices and for cell-based liver therapies. Because hepatocytes are highly differentiated cells residing in a complex microenvironment *in vivo*, they tend to lose hepatic phenotype and function *in vitro*. This paper first reviews traditional culture approaches used to rescue hepatic function *in vitro* and then discusses the benefits of emerging microfluidic-based culture approaches. We conclude by reviewing integration of hepatocyte cultures with bioanalytical or sensing approaches.

## I. INTRODUCTION

The liver is the largest organ in the body and the major site of xenobiotic metabolism. Hence, there has been a strong interest in culturing hepatocytes in order to model liver diseases or predict liver toxicity. Over the years, *in vitro* cultures have sought to recapitulate elements of the native liver microenvironment by creating co-cultures of hepatocytes with non-hepatic cells and by incorporating extracellular matrix (ECM) components.^1,2^

**FIG. 1.**
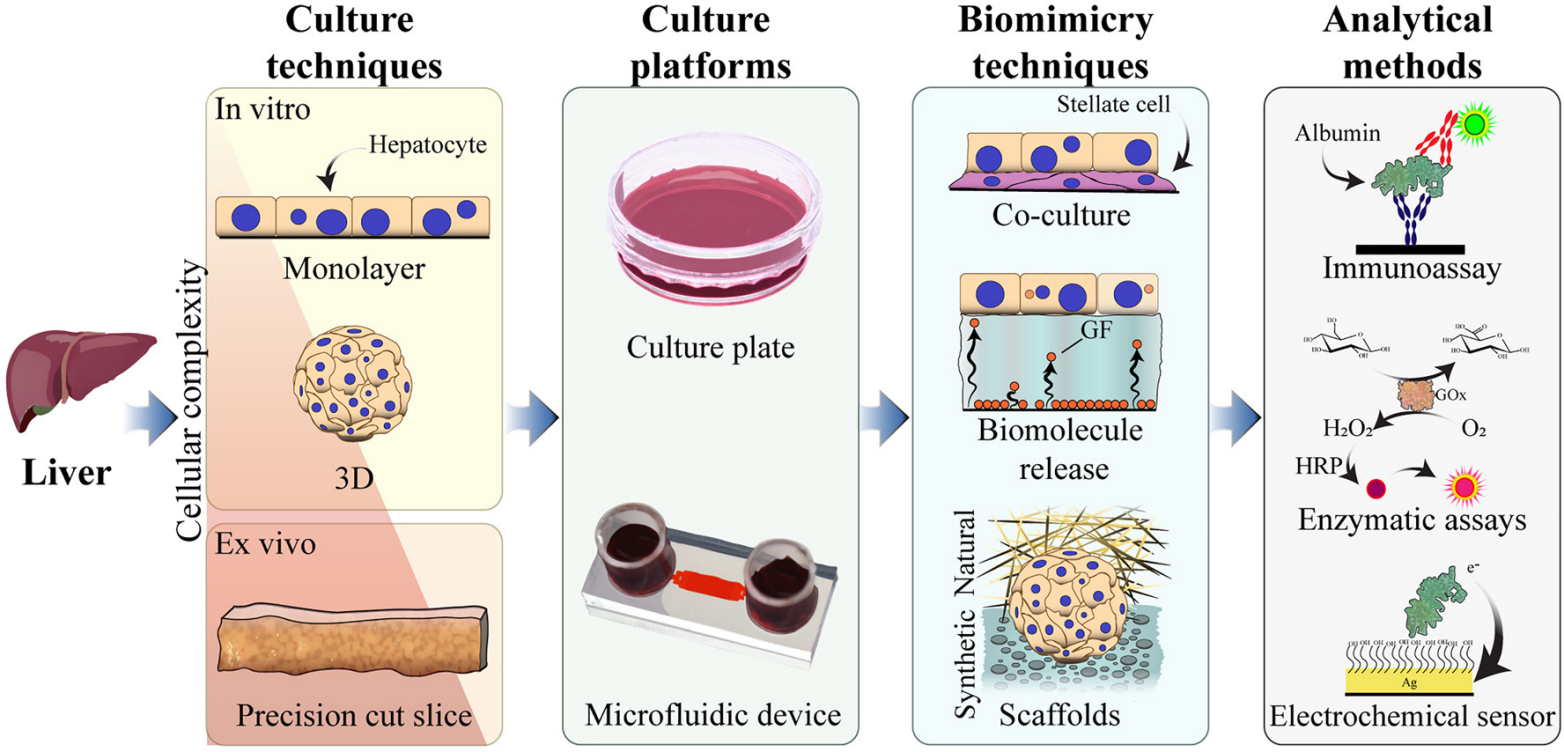
Topics covered in this Review. Along with the conventional culture techniques and platforms for liver tissue engineering, this Review will discuss liver physiology recreated within the microfluidic devices with several biomimicry techniques. Moreover, the possibility of the functional analysis for liver tissues and cells within the microfluidic devices conducted simultaneously with the culture will be examined.

In this Review, we first highlight the essential functions of the liver cells and available cell sources for *in vitro* studies along with traditional methods for cultivation of hepatocytes. Subsequently, we discuss hepatocyte cultures in microfluidic devices and integration of bioanalytical tools into such microfluidic cultures (see Fig. 1).

### A. Morphology

**FIG. 2.**
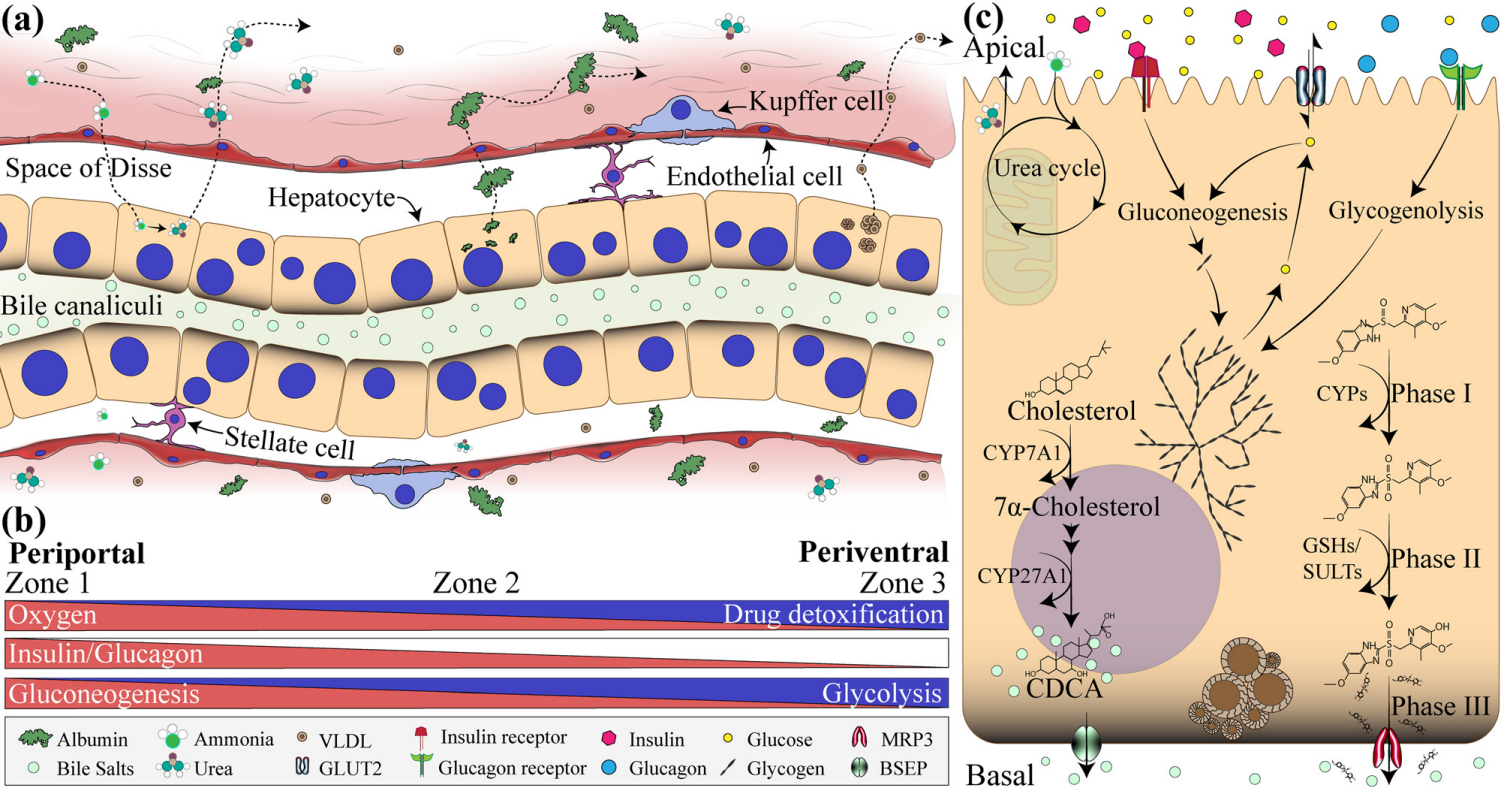
Liver and hepatocyte physiology. (a) Cross-sectional view of the liver sinusoid. Hepatocytes are polarized such that bile acid molecules are released into bile canaliculi from the basal side while proteins are released from the apical side into sinusoidal space lined with endothelial cells. The space between the hepatocytes and the blood vessel is known as the space of Disse, where stellate cells reside. Kupffer cells can be found inside the sinusoids. Biomolecules, such as ammonia, diffuse from the bloodstream toward the hepatocytes, where they are converted into urea and secreted back toward the bloodstream. Albumin and very low-density lipids (VLDLs) are secreted from the apical side of hepatocytes into blood. (b) Zonation of liver sinusoids can be divided into three different groups of hepatocytes based on oxygen and hormonal gradients, so cells can have a specific metabolic function. (c) The hepatocytes engage multiple metabolic pathways, such as drug metabolism (phases I, II, and III), ammonia detoxification through urea cycle, production of bile salts, and glucose metabolism: gluconeogenesis and glycogenolysis in response to insulin and glucagon, respectively.

The liver is in the abdominal cavity and consists of two main lobes with thousands of lobules that contain small ducts that connect the liver to the gallbladder and the small intestine through the bile duct.^3^ Blood supply enters the liver through the oxygen-rich hepatic artery and nutrient-rich portal vein coming from entero-pancreatic circulation and exits via the central vein. The liver is a complex multi-duct system comprised of circa 15 different cell types that work in concert to orchestrate energy metabolism, plasma protein production, detoxification, and removal of foreign particles. Hepatocytes are parenchymal cells of the liver, comprising ∼60% of the total cell mass. These cells are characterized by their three-dimensional (3D) polyhedral morphology and complex apical and basolateral polarization that allows cells to secrete and absorb specific metabolites.^4^ The sinusoidal membrane of the hepatocytes is equipped with microvilli that enhance specific and nonspecific absorption and secretion of plasma proteins in the perisinusoidal space (known as space of Disse) filled with blood plasma [see Fig. 2(a)]. Through their lateral membrane, hepatocytes communicate with and adhere to other hepatocytes via gap and tight junctions. The sinusoidal and lateral membranes together give rise to the basolateral membrane of the hepatocytes. In contrast, the apical membrane is composed of tight junctions between adjacent hepatocytes, forming the bile canaliculi. Other key features of hepatocytes include prominent nuclei, smooth and rough endoplasmic reticulum together with a high number of mitochondria, lysosomes, peroxisome, and ribosomes that sustain the high metabolic and enzymatic activity of these cells.

### B. Metabolism and function of hepatocytes

Metabolic activity of hepatocytes varies depending on their location between the portal vein, hepatic artery, and the central vein [see Fig. 2(b)].^5,6^ Hepatocytes in the periportal (PP) region or zone 1 are exposed to oxygen and nutrient-rich blood. As a result, they exhibit high levels of gluconeogenesis, lipid metabolism, fatty acid oxidation, and ammonia detoxification.^6^ On the other hand, hepatocytes located near the central vein, also known as perivenous (PV) region (zone 3), are exposed to oxygen- and glucose-poor blood, so high levels of glycolysis and xenobiotic metabolism are observed.^7^ Hepatocytes in the intermediate zone (zone 2) can modulate their metabolic function preference based on the oxygen, nutrients, hormones, and cytokine gradients.^3,8^

#### 1. Glucose and lipid metabolism

The liver receives and senses pancreatic hormones (e.g., insulin and glucagon) and acts as a glucostat of the body. In the postprandial state, the pancreas senses high levels of glucose and secretes insulin into portal circulation, which is then sensed by the hepatocytes in the liver. In response, the hepatocyte upregulates glucose consumption and its storage as glycogen (glycogenesis).^9,10^ During fasting, when glucose levels decrease, the pancreas produces glucagon, which, in turn, stimulates hepatocytes in the liver to release stored glucose into the bloodstream. Glucose is produced either by breaking down the stored glycogen (glycogenolysis) or made *de novo* in the process of gluconeogenesis.^11–13^ In order to store glucose as much as possible during the postprandial state, hepatocytes use adenosine triphosphate (ATP) molecules generated through glycolysis as their primary source of energy.^14^ Meanwhile, during fasting and normal metabolism, energy molecules are obtained from oxidation of pyruvate and acetyl-CoA in the Krebs cycle.^11^ Dysregulation in glucose metabolism is generally caused by insulin resistance or type 2 diabetes in which insulin inefficiently suppresses glycogenolysis and gluconeogenesis. As a result, glucose is released into the bloodstream even at normo- and hyperglycemic levels.^10^

Apart from glucose, the liver can store energy in the form of fatty acids (FA) and triacylglycerols (TAG).^11,15,16^ The abundant intracellular glucose in the hepatocytes during the post-prandial period gives rise to pyruvate via glycolysis that is later converted into acetyl-CoA. Through fatty acid synthase (FAS), acetyl-CoA is converted into *de novo* FAs that can be esterified into TAG or cholesterol esters and stored in lipid droplets or secreted into very low-density lipoprotein (VLDL). The abnormal accumulation of lipids or steatosis in the liver is known as nonalcoholic fatty liver disease (NAFLD), and it can evolve into nonalcoholic steatohepatitis (NASH) characterized by chronic liver inflammation and hepatocyte cell death.^10,13,17–19^

#### 2. Bile and urea production

Bile is produced in the liver and secreted into the small intestine to aid lipid digestion. Bile is characterized as a mixture of inorganic electrolytes, glucose, hormones, and principally lipids and bile acids.^20^ Cholesterol is a starting material from which hepatocytes synthesize primary bile acids: cholic and chenodeoxycholic acids (CDCA), through cytochrome P450 (CYP) 7A1 and CYP27A1 from the bile acid synthetic pathway.^21–23^ The basolateral membrane of hepatocytes is equipped with different ATP binding cassettes (ABC), such as bile salt export pump (BSEP), multidrug resistance protein 1 (MRP1), MRP2, MRP3, and ABCG5/G8 for excretion of bile acids, lipophilic cationic drugs, non-bile acid organic anions, phospholipids, and cholesterol. These compounds are excreted into the bile canaliculi and transported out of the liver by osmotic forces of bile acids. Lipids and bile salts serve as detergent to facilitate absorption and transport into the blood stream of lipids, nutrients, and vitamins.

Ammonia is a waste product of protein catabolism, especially from dietary protein and muscle turnover that diffuses freely into the bloodstream. At physiological levels, ammonia helps to stabilize pH and serves as a nitrogen source for glutamine synthesis.^24,25^ Excess ammonia is removed by periportal hepatocytes through a detoxification process called the urea cycle,^26,27^ where urea is created and released into bloodstream and then converted into urine by the kidneys.^28,29^

#### 3. Cytochrome P450 family and other enzymes involved in detoxification

Hepatocytes play a central role in detoxification of endogenous and exogenous chemicals that need to be metabolized or eliminated from the organism to minimize injury. Drugs and xenobiotics are metabolized mostly in the perivenous zone (zone 3), where cells are rich in drug metabolizing enzymes (DMEs) and transmembrane drug transporters.^30^ As shown in Fig. 2(c), drug metabolism can be described in three phases.^31^ Phase I consists of CYPs enzymes that oxidize xenobiotics through mono-oxygenation, such as CYP1A2 and CYP3A.^32^ In phase II, the oxidized xenobiotic compound is conjugated either with glucuronic or sulfuric acid by glutathione S-transferase (GST) or sulfotransferase (SULT) enzymes, respectively, to increase hydrophilicity, easing its secretion through bile and urine.^13,32,33^ Finally, phase III involves the use of transporters, such as MRP3, P-glycoprotein (P-pg) or anion transporting polypeptide 2 (OATP2), to secrete the conjugated compounds out of the hepatocytes.^31^ However, some xenobiotics could be metabolized into bioactivated compounds with strong toxicological effects.

#### 4. Production and secretion of plasma proteins

The liver accounts for the synthesis of more than 50% of the serum proteins that ease the transport of nutrients, fatty acids, proteins, metal ions, drugs, and metabolites in blood.^34^ Most common hepatic serum proteins are albumin, transferrin, and transthyretin.^35,36^ Moreover, albumin has been attributed to have antioxidant function by scavenging reactive oxygen species and contributes to colloid oncotic pressure.^37,38^ Production of albumin by the liver or *in vitro* cultures of hepatocytes is commonly monitored to assess hepatic function in clinical and research studies.

## II. CULTIVATION OF HEPATOCYTES

### A. Sources of hepatocytes

As noted earlier, there is a strong interest in using hepatocyte cultures for predicting drug toxicity or modeling liver diseases. The hepatocytes for these studies come from animal models or from leftover human liver tissue.

There are well-established protocols involving cannulation and collagenase perfusion through the liver to isolate primary hepatocytes from various animal models, of which rodents are most commonly used.^39^ There are, however, concerns of interspecies differences in expression/induction of biotransformation enzymes.^40^ Therefore, human hepatocytes are desirable for testing drug toxicity.

Human hepatocytes are available from cadaveric livers that do not qualify for transplantation or from liver resections/biopsies.^41^ Once the tissue is dissected, hepatocytes become vulnerable to ischemia and decay quickly. Regardless of how the tissue is preserved and digested, cellular yield is poor, and a significant decrease in phase I and II metabolizing enzymes has been reported.^39,42–44^

Another promising approach is to employ chimeric mice with humanized livers. These immunosuppressed mice are engineered such that native hepatocytes are unable to survive upon accumulation of endogenous toxic tyrosine metabolite intermediates or through proteolytic damage by overexpressed serine protease. Thus, when healthy human hepatocytes are inoculated into this mouse model, they repopulate the liver with a replacement index (RI) of 70%–90%.^45^ During the proliferation of human hepatocytes, architectural features, such as bile canaliculi and sinusoids, and hepatic functions, such as CYP's and albumin secretion, are restored.^46^ Subsequently, human hepatocytes may be harvested from mice using well-established collagenase digestion protocols mentioned above. Similar strategies of humanizing liver are being taken to the next level in pigs.^47^ In our opinion, chimeric animal models represent the best source of human hepatocytes, although there are concerns about incomplete eradication of and contamination by mouse hepatocytes.

In addition to primary hepatocytes, immortalized cell lines have also been used for studies of hepatic function and toxicity. The cells derived from healthy liver tissues, such as AML12 or THLE-2, and the hepatoma cell lines, HepG2 or Huh-7, are widely utilized in studying liver metabolism.^48–50^ HepaRG cells are also derived from hepatocarcinoma but possess several characteristic features of primary hepatocytes. This cell line has been used as an alternative to primary hepatocytes in studying liver metabolism.^51–53^

Pluripotent stem cells (PSCs) represent another promising source of hepatocytes and have been differentiated into hepatocyte-like cells. Maturity and metabolic activity of the hepatocyte-like cells remain a work in progress;^54–56^ however, given their unlimited capacity for self-renewal, hPSCs hold tremendous promise as a source of hepatocytes.

### B. Non-microfluidic cultures of hepatocytes

**FIG. 3.**
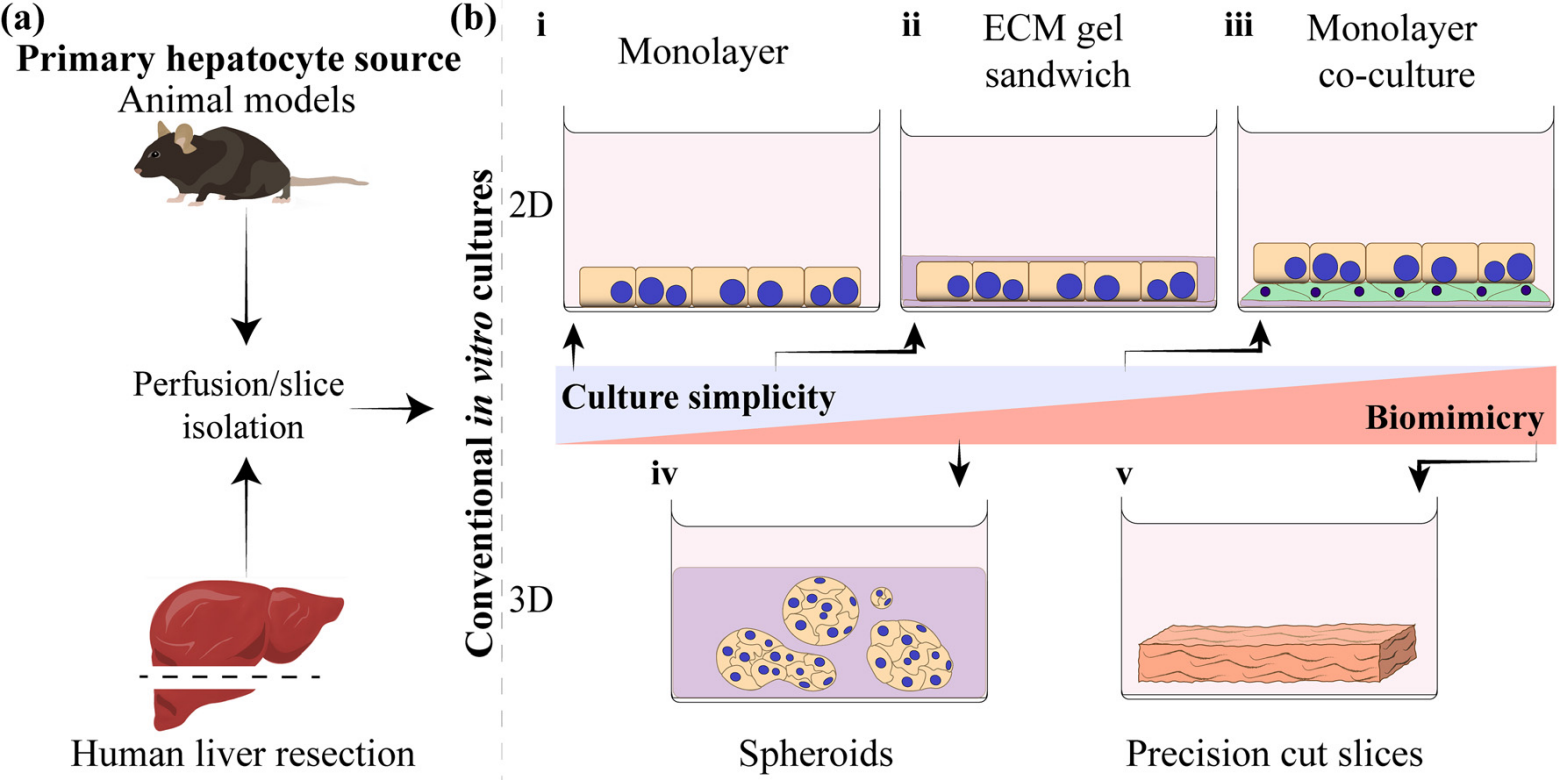
Sourcing and culturing primary hepatocytes. (a) Primary hepatocytes can be isolated from animal models, such as rodents, or from human liver resections. (b) Hepatocyte culture approaches categorized by degree of complexity or biomimicry. Simple 2D hepatocyte cultures (i) are not very functional. This led to the development of ECM gel cultures (ii), co-cultures with non-parenchymal cells (iii), hepatocyte spheroids in ECM gel, (iv) and have culminated in precision cut slices (v).

As noted above, various cellular and molecular components of the liver microenvironment are incorporated into culture systems to ensure long-term maintenance of functional hepatocytes *in vitro*. We wanted to first provide a brief overview of the traditional hepatocyte culture methods in the section below, and then later in this Review, discuss how these traditional approaches are being adapted for microfluidic cultures. Conventional methods for extending hepatocyte maintenance *in vitro* are described below (see Fig. 3).^57–59^

#### 1. Hepatocytes in ECM gels and other 3D cultures

The ECM is a complex network of proteins and polysaccharides that serve as scaffolding for cells and signals. Collagens I and IV are the most abundant molecular elements comprising the ECM of the liver. Collagen I is prevalent in healthy human liver while collagen IV is dominant in fibrotic liver; however, for reasons of cost and accessibility, collagen IV has been used more frequently for culturing hepatocytes.^60–62^ One of the early uses of the ECM was reported by Dunn *et al.* who demonstrated long-term maintenance of hepatocytes sandwiched between layers of gel composed of collagen IV.^63,64^ In conceptually similar studies, hepatocytes placed into Matrigel expressed epithelial markers and maintained high levels of hepatic function.^65^

Heparin is another ECM element abundant in the liver.^66,67^ Borrowing from the collagen gel sandwich format discussed above, Foster *et al.* created hepatocyte cultures sandwiched between heparin-containing hydrogel.^68^ The albumin and urea secretion were similar between collagen and heparin double layers, but activity of several CYPs was significantly higher for cells cultured on heparin gel. Moreover, bioactivity of the heparin gel was leveraged for incorporation of hepato-inductive morphogen and hepatocyte growth factor (HGF), which further enhanced hepatic function.

In addition to ECM gel-sandwich cultures, hepatocytes can be initially mixed with ECM solutions to create 3D microtissue such as gel fibers and beads.^69–72^ Geometry of the microtissue ensures that embedded single cells or cell aggregates receive sufficient nutrients and oxygen.^69^ In other approaches, a mixture of ECM fibers and hepatocytes was 3D printed into desirable structures.^70,73–76^

#### 2. Random and micropatterned co-cultures of hepatocytes and non-parenchymal cells

As noted above, incorporation of hepatocytes into ECM gel enhanced hepatic phenotype and function. Another strategy for phenotype enhancement has been to co-culture hepatocytes with nonparenchymal liver cells. Some of the early studies demonstrating enhanced hepatic function in the presence of nonparenchymal cells were carried out in mid-1980s by Guguen-Guillouzo and colleagues.^77,78^ More recently, Bhandari and colleagues showed that the presence of 3T3 fibroblasts led to enhanced viability and increased markers of hepatic function, including albumin and 7-ethoxyresorufin O-dealkylation (EROD) activity.^79^ Cho *et al.* demonstrated that primary hepatocytes co-cultured with 3T3-J2 fibroblasts had similar production of albumin compared to hepatocytes cultured in collagen gel sandwiches.^80^ Additional studies demonstrated that the presence of hepatic stellate cells enhanced function of hepatocytes by showing more prolonged and elevated secretion of albumin.^81–83^ While the degree of enhancement appears to vary depending on specifics of the experimental system, there is clear evidence that hepatocytes benefit functionally and phenotypically from co-cultures with non-parenchymal cells.

The next stage in the development of hepatocyte co-cultures came in the late 1990s when Bhatia and colleagues proposed to use photolithography to create a pattern of collagen islands and then demonstrated selective adhesion of hepatocytes to these islands.^84,85^ Subsequently, secondary cells could be added in to fill spaces between islands of hepatocytes, thus creating a micropatterned hepatocyte-stromal cell co-cultures. These co-cultures were first used to study the effects of homotypic and heterotypic interactions in rodent hepatocyte cultures,^86^ and later extended to human hepatocyte cultures by Khetani *et al.*^87,88^ Jeong and colleagues tested the importance of paracrine signals by comparing mono-cultured primary rat hepatocytes vs direct or indirect co-cultures with mouse fibroblasts. Direct co-cultures led to the highest hepatic function, which was clearly represented with the highest secretion of albumin and urea synthesis, followed by fibroblast-conditioned media with mono-cultures showing lowest function.^89^

#### 3. Spheroid cultures of hepatocytes

There has been considerable interest in 3D or spheroid cultures of hepatocytes. The 3D format maximizes cell–cell contacts and provides more physiological environment compared with 2D cultures.^69,70^ Cellular aggregates for 3D cultures may be generated by various means, including rotational bioreactors, microwells, gel-embedment, or polymeric scaffolds.^90–92^ For example, Chang *et al.* used rotating wall vessel to culture primary mouse hepatocytes in a spheroid format and demonstrated that hepatic phenotype and gene expression were upregulated in the 3D format compared to monolayer cultures.^93^ Wong *et al.* used microwells to form primary hepatocytes into 3D constructs for disease modeling.^94^ The size of the hepatocyte spheroid is an important parameter as was demonstrated by Glicklis and colleagues, who compared viability, oxygen transport, and albumin secretion of spheroids ranging from 100 to 600 *μ*m in diameter. The conclusion was that smaller spheroids were associated with higher viability and functionality of hepatocytes.^95^

#### 4. Precision-cut liver slices—Cultivation of intact liver tissue

One way to recapitulate cellular interactions and ECM components present *in vivo* is to culture intact pieces of liver. Precision-cut liver slices (PCLS) represent one example of intact liver tissue cultures.^96–98^ To ensure that nutrients are delivered uniformly, tissue is sliced to <450 *μ*m using a vibratome or a Krumdieck slicer.^99–101^ Liver slices have been extensively used for drug metabolism and modeling, such liver diseases as liver fibrosis and NAFLD.^98,102^ Despite their benefits, liver slices have drawbacks, chief among which is decay in viability and hepatic function within 5 days of culture.^96,103–105^

#### 5. Comparison of hepatic function across different culture systems

Sections above described various methods for culturing hepatocytes and maintaining hepatic function. However, what constitutes a high level of hepatic function and how does function compare across culture formats? To answer this question, we reviewed hepatocyte culture literature and tabulated data for albumin and urea, the most commonly used indicators of hepatic function (see Table I). Because there is no standard way of reporting these values, we standardized them based on experimental details provided for these studies. Given that hepatic function may vary depending on species, we chose to focus on rat hepatocytes as these cells appear more commonly in the literature. Summary of these data presented in Table I points to micropatterned co-cultures eliciting levels of albumin and urea production among non-microfluidic cultures. Microfluidic hepatic cultures described later in this Review also elicit high levels of hepatic function.

**TABLE I.** Comparison of albumin and urea production across different hepatocyte culture formats. Unless indicated otherwise, function is reported for primary rat hepatocytes.

	Culture duration (day)	Albumin level (*μ*g/1 × 10^4^ cells/day)	Urea level (*μ*g/1 × 10^4 ^cells/day)
Monolayer culture	12^57^	0.014^59^	0.6^59^
ECM gel	42^44^	0.638,^58^ 0.72^63^	125^68^
Micropatterned co-culture	27^83^	3,^83^ 3.8^86^	7.2,^83^ 192^86^
Liver slices	15^96^	0.087^103^	N/R
Microfluidic monolayer	21^108^	1,^109^ 3^108^	2^110^^,^^a^
Microfluidic spheroid cultures	21^109^	1.35,^109^ 0.08^111^^,^^a^	320^112^
Microfluidic slices	3^97,105,113^	N/R	N/R

^a^ Primary human hepatocytes.

For comparison, hepatic albumin and urea production *in vivo* (in rats) is 1.08 *μ*g/1 × 10^4^cells/day^106^ and 2.3 *μ*g/1 × 10^4^cells/day,^107^ respectively, which means that the function observed in some of the best hepatic culture systems is physiological or even supraphysiological. It is worth noting that hepatic function is complex, and albumin and urea production reflect two facets of this function—protein synthesis and nitrogen metabolism. A more comprehensive analysis of hepatic function across culture platforms should include analysis of enzyme expression and function; however, such analysis is less commonly performed and could not be tabulated here.

## III. CULTIVATION OF HEPATOCYTES IN MICROFLUIDIC DEVICES

Microfluidic devices have emerged as an important tool for cultivation of hepatocytes. Such devices offer a number of benefits, such as a small number of cells needed for cultivation, precise control over composition and flow rate, the possibility of generating gradients that mimic liver zonation, and the opportunity to integrate sampling or bioanalysis units alongside cells.

While microfluidic devices may be fabricated by various methodologies and may be comprised of different types of materials, these devices are most-commonly composed of poly(dimethyl-siloxane) (PDMS) and fabricated by soft-lithography. PDMS continues to be used widely because of its excellent biocompatibility and gas permeability, the latter property being particularly important for culturing oxygen-consuming cells, such as hepatocytes. One of the earliest examples of long-term hepatocyte cultures in microfluidic devices was provided by Kane *et al.*, who designed 8 × 8 microfluidic wells that can support micropatterned primary rat hepatocytes for co-culture with 3T3-J2 fibroblasts. With constant perfusion of culture media into the microfluidic device, high levels of albumin and urea production were observed up to 1 month.^114^ Other early studies demonstrating benefits of microfluidic cultures include the work from the Luke Lee lab who incorporated elements of fenestration and sinusoidal architecture into microfluidic cultures of hepatic cells.^115,116^ Sections III A–III F go into greater detail on different aspects of microfluidic cultivation of hepatocytes.

### A. Small volume effects contribute to enhanced function of hepatocytes in microfluidic devices

**FIG. 4.**
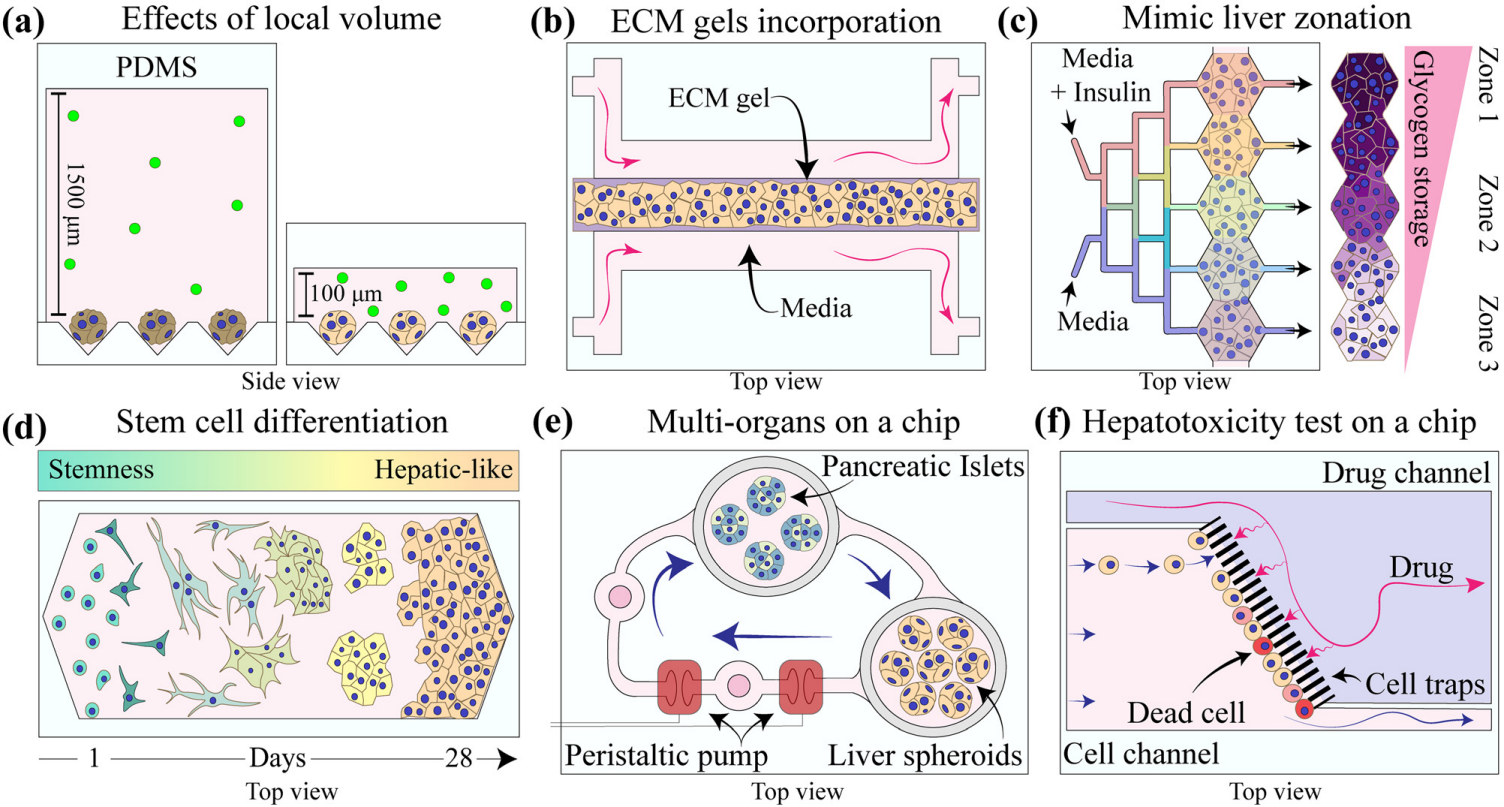
Applications of microfluidic devices for culturing hepatocytes. (a) Confinement of hepatic cultures in microfluidic devices in the absence of convection increases accumulation of endogenous growth factors (concept from Ref. 109). (b) Imbedding of hepatocytes into collagen gel inside a microfluidic while delivering nutrients through an adjacent channel improves hepatic function (concept from Ref. 60). (c) Liver zonation recreated in a microfluidic device by generating a gradient of hormones or drug along the length of the device (concept from Ref. 117). (d) Microfluidic devices improve stem cell differentiation into hepatocyte-like cells. (e) Multi-organ interactions using microfluidic devices with built-in peristaltic pumps (concept from Ref. 118). (f) A microfluidic device for unidirectional drug exposure and testing hepatoxicity (concept from Ref. 119).

Small volumes of microfluidic devices provide interesting and unexpected benefits for culturing hepatocytes [see Fig. 4(a)].^113^ Our team noted that hepatocytes placed into a microfluidic channel, in the absence of flow but in the presence of sufficient nutrient supply, remained functional for up to 21 days as monolayer cultures. Hepatocytes cultured under identical conditions in standard (large volume) format lost phenotype and function within 7 days.^108^ We determined that hepatic phenotype enhancement in microfluidic channels was due to greater production and accumulation of endogenous growth factors including HGF, EGF, and IGF. For example, adding HGF inhibitors into culture media resulted in a rapid loss of hepatic function in microfluidic devices. In addition to monolayer hepatocyte cultures, we demonstrated that microfluidic confinement improved function of hepatocyte spheroids.^109^

### B. Microfluidic hepatic cultures enhanced by incorporation of ECM gels

As discussed earlier, ECM gels have been used widely to improve phenotype and function of hepatocytes. It, therefore, stands to reason that ECM components will benefit microfluidic hepatocyte cultures as well [see Fig. 4(b)].^112^ Hegde and colleagues developed a microfluidic version of a collagen gel sandwich where hepatocytes are cultured between two vertically positioned chambers filled with collagen I gel. Hepatocyte cultures with or without flow were compared, and those exposed to flow were found to have higher levels of albumin and urea synthesis. In addition, a better defined bile canaliculi network was formed under dynamic culture conditions compared to static cultures.^60^ In another study, Lu *et al.* incorporated decellularized liver matrix (DLM) with methacrylated gelatin into a microfluidic device and demonstrated improved stability and functionality of hepatocytes.^120^

### C. Mimicking liver zonation in microfluidic hepatic cultures

The oxygen, nutrients, and hormonal gradients generated along the hepatic sinusoid induce different levels of enzymatic activity in hepatocytes residing along the PP to PV axis. This phenomenon is called liver zonation (see metabolic pathway section). Several microfluidic approaches have aimed to reproduce this zonation *in vitro* [see Fig. 4(c)].^121–123^ For example, Tsukada and colleagues designed a microfluidic device with an oxygen gradient along the hepatic monolayer culture by placing an air-gas channel beneath the PDMS membrane.^122^ Real-time measurements of oxygen were used to reveal differences in hepatic metabolism and the appearance of three distinct zones within 5 days of culture under gradient conditions. Tonon *et al.* reported that hepatocyte metabolic zonation was retained for up to 17 days when hepatocytes were cultured in a gas permeable PDMS chamber where a steady oxygen gradient was created by controlling continuous media infusion.^124^ Hepatic zonation in microfluidic devices can also be achieved when cultured in a hormone gradient. Yarmush and colleagues coupled a gradient generator unit to a culture chamber where hepatocytes are exposed to five different hormonal or xenobiotic concentrations, including insulin and acetaminophen.^117,123^ Cells revealed a gradient in metabolism with glycolysis and gluconeogenic observed in cells residing on the opposite sides of the chamber due to the hormonal gradient generator unit.^123^

### D. Hepatic differentiation of stem cells in microfluidic devices

As discussed earlier in this Review, there are significant challenges associated with sourcing of human hepatocytes. PSCs proliferate indefinitely and may be differentiated into any adult cell types, including hepatocytes. Differentiation protocols are typically carried out in multi-well plates where cells are exposed to different inductive cues (growth factors or inhibitors) at specific temporal windows [see Fig. 4(d)].^125–129^ While robust protocols for hepatic differentiation of hPSCs have been established, these protocols require large volumes and expensive reagents. Furthermore, resultant hepatocytes have lower levels of enzymatic activity compared to adult hepatocytes.^130,131^

Microfluidic devices allow one to decrease the use of reagents and the cost of differentiation protocols. In one example, Giobbe *et al.* directed differentiation of PSCs into hepatocytes in a microfluidic device. A key element of this study was the finding that media exchange frequency played an important role in the differentiation protocol. Exchanging the volume of the device twice per day resulted in better pluripotency and better differentiation efficiency compared to more frequent volume exchanges.^132^ This observation points to the importance of accumulating endogenous signals in a microfluidic culture format and parallels our team's findings on this topic.^108,109^

### E. Microfluidic co-cultures of hepatocytes and non-parenchymal cells

We previously discussed that the function of hepatocytes in a culture dish may be improved by creating random or micropatterned co-cultures with nonparenchymal cells with improvement being attributed to juxtacrine and paracrine interactions between the cell types. Heterotypic liver cell cultures have also been implemented inside microfluidic devices. For example, Kane and colleagues created a microfluidic platform in which patches of hepatocytes are cultured in a monolayer of nonparenchymal cells for more than 20 days with a stable albumin and urea secretion.^114^ A more recent and sophisticated culture system was reported by Jang and colleagues who attempted to recapitulate the architecture of the Space of Disse by placing liver sinusoidal endothelial cells, Kupfer cells, and stellate cells on a porous membrane with primary hepatocytes imbedded in ECM gel on the opposite side of the membrane.^133^ These complex microfluidic liver cultures had 14-fold higher albumin production than traditional collagen gel sandwich cultures of hepatocytes.

As noted earlier, liver is the metabolic hub of the body, which communicates with the pancreas, gut, and gallbladder via secreted hormones, lipids, and proteins. For example, glucose uptake or release in the liver is regulated by pancreatic hormones that are released into blood stream.^10^ Microfluidic systems allow one to mimic some of the anatomical/physiological interconnections between organs [see Fig. 4(e)].^134–136^ In one example, Leclerc and colleagues first established primary rat hepatocytes and primary rat islets cultures in two independent devices, and then interconnected these devices using tubing and a peristaltic pump.^137^ Hepatocytes were shown to maintain function in this system for 7 days although islet function appeared to decay. In a conceptually similar approach, Bauer *et al.* cultured hepatic spheroids in a microfluidic chamber positioned downstream of the human pancreatic islet chamber. Physiological crosstalk between both the organ models was achieved by recirculation of the media with the aid of an on-board peristaltic pump. The authors demonstrated that this integrated system achieved normoglycemic glucose levels 48 h after a high glucose challenge and that both cell types could be maintained for 13 days in this system.^118^

Absorption, transport, and breakdown of orally administered xenobiotics have been replicated in microfluidic devices to understand drug loading and transport into the liver. Shuler and colleagues designed a plug-in co-culture platform of Veroclear polymers where gut epithelium and primary human hepatocytes were cultured independently.^110^ After tissue maturation, both devices were stacked together and connected through porous membranes, where unidirectional re-circulation by gravity-driven flow was achieved by placing the device in a rocking platform. Hepatocyte functions remained stable for 14 days, and CYPs activity was slightly increased when co-cultured with gut epithelium. In comparison, Groothuis and colleagues co-cultured precision-cut liver and gut slices in a microfluidic device with sequentially perfused chambers.^138^ Aside from improvement of drug metabolization, bile synthesis was downregulated by fibroblast growth factor 15 (FGF15) secreted by the gut epithelium, mimicking liver and gut inter-communication. Other absorptive organ models (e.g., lung and skin) were co-cultured with HepaRG organoids to study xenobiotic metabolism in microfluidic device.^111,139^

### F. Using microfluidic devices to predict hepatotoxicity of drugs

Given the central role of the liver in metabolizing toxicants, it is critical to predict hepatotoxicity during drug development.^140–142^ This motivates the need for culture systems that predict metabolism of novel drugs and injury to the liver. A number of reports described microfluidic devices containing an array of hepatocyte culture compartments where each compartment received a different type or concentration of the drug of interest.^143–148^ A different approach was taken by the team led by Yarmush and Usta who published a series of papers describing the use of the gradient generator to perfuse different concentrations and combinations of drugs into one microfluidic chamber populated with hepatocytes.^123,149–152^ This capability allows us to conserve the amount of material needed for testing.

In addition to studies described above, there has been increasing emphasis on integrating microfluidic automation and hepatic cultures. In one example, Pasirayi and colleagues designed a microfluidic device integrating a gradient generator with 24 chambers containing HepG2 cells.^153^ This array of cell culture chambers was integrated with normally closed valves that prevented crosstalk. Desired microvalves were opened by applying negative pressure for seeding cells or delivering drugs. By controlling the sequence of valve actuation and drug delivery, the authors were able to test multiple concentrations of a model drug, pyocyanin.

Another interesting approach for short-term toxicity tests was reported by Yeon and colleagues, who captured primary human hepatocytes and HepG2 cells in hydrodynamic traps connected to a drug delivery channel [see Fig. 4(f)].^119^ This device was designed to imitate hepatocyte proximity to fenestrated endothelial cells in the liver sinusoid. The hepatotoxicity of several drug types was monitored over the course of 6 h using propidium iodide (PI) for staining dead cells. However, this platform was not suitable for monitoring hepatotoxicity in the longer timeframe due to exposure of cells to continuous flow and associated shear stress.

## IV. INTEGRATION OF BIOANALYTICAL APPROACHES WITH MICROFLUIDIC HEPATIC CULTURES

The most-commonly used approach for evaluating function of microfluidic hepatic cultures is collecting conditioned media and analyzing it off-chip. Such an approach is useful but offers limited temporal resolution and is associated with significant dilution of signals. There is, therefore, a considerable drive to integrate analytical capabilities into microfluidic cultures for on-chip analysis of hepatic function.

### A. Colorimetric assays in microfluidic cell cultures

There is a plethora of commercially available mix-and-read assays for detecting a range of analytes relevant to hepatic function from energy metabolites to urea, albumin, and markers of hepatic toxicity (e.g., AST and ALT). However, these assays are typically carried out in multi-well plates using milliliter volumes.

**FIG. 5.**
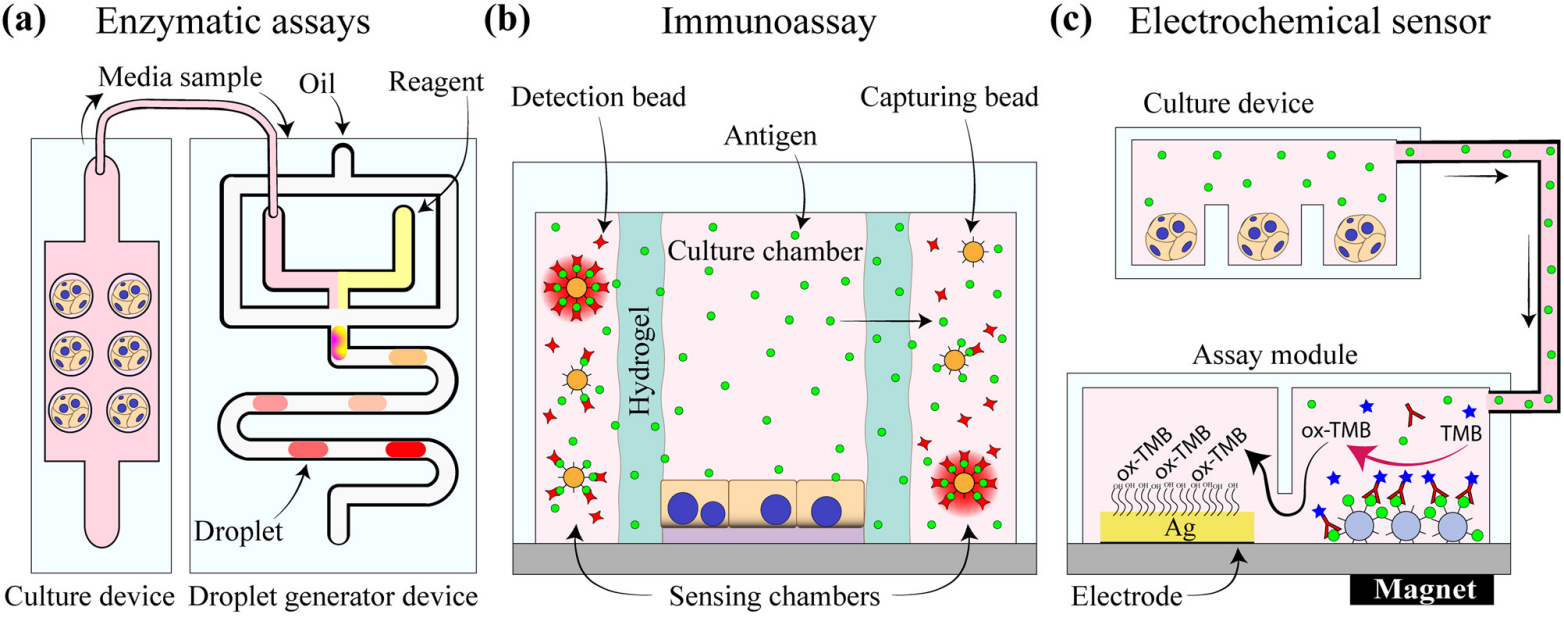
Integration of biosensors into microfluidic hepatic cultures. (a) Microfluidic droplet generator employed for analysis of media conditioned by hepatocytes. Different reactions/bioassays are carried out in a different set of droplets (concept from Ref. 154). (b) A microfluidic hepatocyte culture device integrating channels with hydrogel walls that allow for diffusion of proteins secreted by cells. Bead-based immunoassays are placed into channels flanking the hepatocyte culture chamber and are used for detection of secreted growth factors (concept from Ref. 155). (c) Electrochemical biosensor module coupled to a microfluidic culture device for detection of hepatic biomarkers (concept from Ref. 156).

Our team employed microvalve-enabled microfluidic devices to miniaturize mix-and-read assays such that microliter volumes available in microfluidic cultures could be analyzed. In this study [see Fig. 5(a)], our team demonstrated that a microfluidic analysis module may be connected to a cell culture chamber for absorbance and fluorescence-based analysis of glucose, total bile acids, and cytotoxicity marker LDH.^154^

Other microfluidic-based detection strategies have been used to study metabolism of xenobiotics. Zhang and colleagues reported on the implementation of the EROD activity assay on a microfluidic device that consisted of a culture chamber with three inlets.^145^ They analyzed the conversion of 7-ethoxyresorufin (7-ER) into the fluorochrome resorufin by CYP4501A. The small volume chamber and the optical properties of glass-PDMS devices allowed these researchers to observe kinetics of metabolic breakdown in real-time at the level of single rat hepatocytes.

### B. Immunoassays in microfluidic cell cultures

Immunoassays are widely used for the detection of proteins or peptides. Typically, immunoassays are carried out using benchtop equipment either automated or manual. Valve-enabled microfluidic devices are particularly well-suited for miniaturization and automation of immunoassays.

For example, Son and colleagues developed a microfluidic system where the hepatocyte culture chamber was separated from the sensing chamber by a permeable hydrogel barrier [see Fig. 5(b)]. Growth factor molecules (HGF and TGF-β1) secreted from hepatocytes diffused across the hydrogel barrier and into the sensing chamber where these biomolecules were captured by polystyrene microbeads precoated with specific antibodies. Nanobeads functionalized with fluorescently labeled antibodies were used for the detection of growth factors binding to microbeads.^155^ This system allowed the researchers to detect HGF and TGF-β1 at the site of hepatocytes with minimal dilution. In another example of integration of an immunoassay into hepatocyte cultures, Luan *et al.* also analyzed albumin secreted from cultured hepatocytes in a microfluidic system by using polystyrene beads. This device consisted of one culture chamber for hepatocytes and two inlets that were used for introduction of biotinylated albumin antibodies conjugated to polystyrene beads and fluorescently labeled secondary antibodies, respectively.^157^

### C. Electrochemical biosensors in microfluidic cell cultures

Electrochemical biosensors often offer simpler and more robust alternatives to optical biosensors. There are multiple examples of electrochemical biosensors being integrated into hepatocyte cultures in microfluidic devices. For example, Riahi *et al.* described a microfluidic platform with an on-board sensor for continuously monitoring albumin and transferrin secreted by hepatocytes over the course of 5 days. These proteins were captured on magnetic beads inside a microfluidic device, then labeled with HRP-carrying secondary antibodies and finally were exposed to color reagent molecule, TMB. The oxidation of the latter could be electrochemically detected [see Fig. 5(c)]. These researchers demonstrated a limit of detection that was 10-fold lower than the conventional off-chip test (ELISA), and the overall procedure was fully automated.^156^

Our team has employed electrochemical aptamer-based sensors for the detection of proteins secreted by injured hepatocytes in microfluidic devices. In this study, hepatocytes were co-cultured with stellate cells in a microfluidic device that also contained miniature electrodes functionalized with TGF-β1-sepcific aptamer molecules. These co-cultures were injured by exposure to alcohol, and the production of TGF-β1 was monitored electrochemically. Using this sensor-integrated microfluidic device, we established that, upon alcohol insult, TGF-β1 originated from hepatocytes and then stimulated activation of stellate which, in turn, began producing TGF-β of their own.^158^ Thus, microfluidic devices integrated with biosensors may be used to establish cause-consequence relationships in multi-cellular crosstalk.

Impedance represents another commonly used electrochemical sensing method. Shin *et al.* demonstrated the integration of impedance-based biosensors for albumin and glutathione-S-transferase-alpha (GST-α) into spheroid culture of hepatocytes. This microfluidic system was automated to regenerate biosensors between measurements and extend the use of sensors during cell culture.^159^

## V. ADVANTAGES AND LIMITATIONS OF MICROFLUIDIC HEPATIC CULTURES

While microfluidic liver cultures have evolved over the past decade to become more physiological, there are challenges and questions that need to be addressed for further adaption of microfluidic devices by the biomedical researchers. Fabrication and operation of microfluidic devices requires a significant level of expertise and represents a major hurdle to adoption and dissemination. While it is true that simpler devices may be prototyped rapidly and inexpensively using thermoplastic materials, such device may be challenging to automate with valves and integrate with bioanalytical modules. Therefore, fabrication of automated microfluidic devices continues to involve elastomers, namely, PDMS, which makes the process of device assembly labor-intensive and not particularly scalable. Alternative materials and processes for rapid and scalable fabrication of complex multi-layer devices will represent a major breakthrough for microfluidic hepatic cultures specifically and for microphysiological systems as a field.

There continues to be a healthy debate about balancing physiological complexity and practicality of microfluidic hepatocyte cultures. One could argue that the ideal microfluidic liver system ought to mimic a structure of a liver sinusoid with a perfusable blood vessel lined by endothelial cells with Kupffer cells residing inside the blood vessel and stellate cells situated outside the blood vessel next to hepatocytes. While such systems are being fabricated, they are prohibitively complex to make in sufficient numbers for testing multiple experimental conditions in multi-factorial experiments. Furthermore, increased cellular complexity of liver-on-chip devices brings to the fore the question of cell sourcing and cell quality. Therefore, biologic mimicry and complexity of microfluidic liver cultures need to be balanced with the practicality of setting up experiments with multiple controls and biological replicates. We would, therefore, argue that microfluidic liver cultures need to be “complex enough” to address a specific biomedical question. For example, short term hepatotoxicity is likely modeled reasonably well with hepatocyte cultures whereas liver fibrosis models should, in addition to hepatocytes, include stellate cells.

Despite some of these challenges, we view the future of liver-on-chip devices as bright and hopeful. In particular, we envision that such devices may be populated with patient cells or liver tissue and may supplement or possibly supplant animal models for preclinical drug testing and disease modeling. Furthermore, given that the liver is integrated with several other organs, for example, the gut and the pancreas, we foresee continued development of multi-organ systems for modeling origins and progression of liver diseases.

## VI. CONCLUSIONS

Microfluidic devices hold considerable promise for the fields of disease modeling, preclinical testing, and drug discovery. Microfluidics allow one to recreate liver zonation *in vitro* by exposing cells to gradients of hormones, drugs, or oxygen. Microfluidic systems may also be populated with cells representing multiple organs that communicate with liver to recreate aspects of inter-organ communication present *in vivo*. Moreover, microfluidic devices may also be integrated with biosensors for on-chip sampling and analysis of cell function. When integrated with microvalves, microfluidic devices may be fully automated and used to perform multi-step washing and media exchange protocols. Moving forward, we envision that microfluidic devices will become increasingly complex and capable of manipulating and measuring hepatocellular microenvironment. We are particularly excited about the use of microfluidic devices for personalized medicine applications where patient-specific liver, and other cell types could be cultured and exposed to therapy candidates. Another promising direction is microfluidic cultures of intact liver tissue. Such microfluidic cultures may represent a simpler alternative to precision-cut liver slices and may be used as patient avatars for personalized therapy selection.

## Data Availability

Data sharing is not applicable to this article as no new data were created or analyzed in this study.
